# Influence of academic stress and school bullying on self-harm behaviors among Chinese middle school students: The mediation effect of depression and anxiety

**DOI:** 10.3389/fpubh.2022.1049051

**Published:** 2023-01-06

**Authors:** Hui Chen, Huijuan Guo, Haiyan Chen, Xia Cao, Jiali Liu, Xianliang Chen, Yusheng Tian, Huajia Tang, Xiaoping Wang, Jiansong Zhou

**Affiliations:** ^1^Department of Psychiatry, National Clinical Research Center for Mental Disorders, The Second Xiangya Hospital of Central South University, Changsha, Hunan, China; ^2^Centers for Disease Control and Prevention, Changsha, Hunan, China; ^3^Health Management Center, Health Management Research Center of Central South University, The Third Xiangya Hospital, Central South University, Changsha, Hunan, China

**Keywords:** school bullying, academic stress, suicide attempt, non-suicidal self-injury, depression

## Abstract

**Objective:**

The purpose of this study was to explore the relationship between academic stress, school bullying and self-harm behaviors among Chinese middle school students and to further explore the impact of anxiety and depression on this relationship.

**Methods:**

The students (aged 12–16 years) in a middle school in Changsha city were invited to respond to a questionnaire through an online platform. The Patient Health Questionnaire-9 (PHQ-9) and Generalized Anxiety Disorder-7 (GAD-7) were used to assess the severity of anxiety and depressive symptoms, respectively. The experience of being bullied, academic stress, and self-harm behaviors were assessed using several questions on the basis of previous studies.

**Results:**

A total of 1,313 middle school students completed the study, and 3.40% and 4.10% of them reported suicide attempts (SAs) and non-suicidal self-injury (NSSI), respectively. Univariate logistic regression analysis revealed that anxiety symptoms (OR = 1.23, 95% CI: 1.18–1.28; OR = 1.25, 95% CI: 1.19–1.31), depressive symptoms (OR = 1.20, 95% CI: 1.16–1.25; OR = 1.26, 95% CI: 1.20–1.31), school bullying (OR = 3.8, 95% CI: 2.11–6.89; OR = 2.76, 95% CI: 1.39–5.47), and academic stress (OR = 2.20, 95% CI: 1.27–3.80; OR = 3.80, 95% CI: 1.20–7.25) were common factors of NSSI and SAs. In addition, depressive symptoms showed a mediating effect on the association of school bullying and academic stress with SAs or NSSI, and anxiety symptoms showed a mediating effect on the association of school bullying and academic stress with NSSI only.

**Conclusion:**

Appropriate strategies are needed to reduce academic pressure and prevent school bullying. Meanwhile, negative emotions such as depression and anxiety should be evaluated and intervened in to prevent self-harm behaviors among middle school students.

## 1. Introduction

Self-harm behaviors were defined as intentional acts of self-destruction, including intentional cutting and poisoning, regardless of suicidal intent or any other motivation, which has become an important public health problem among adolescents (1). Based on whether adolescents have suicidal intentions, self-harm was classified a suicide attempt (SA) or non-suicidal self-injury (NSSI) (2). A recent study in Norway showed that 16.2% of adolescents (13–15 years) had engaged in self-harm behaviors (3). A meta-analysis reported that the overall prevalence of self-harm among Chinese adolescents was 22.37% (4). SAs and NSSI differ in terms of intention and lethality, but they also share many risk factors, including sociodemographic and educational factors, negative life events, family adversities, and mental illness factors; furthermore, NSSI was found to be strongly predictive of SAs (5). Both SAs and NSSI greatly impact the physical and mental health of adolescents and are associated with an increased risk of suicide, which places a greater burden on society. Therefore, it is crucial to identify adolescents who are most prone to self-harm and to formulate and implement precise prevention and intervention strategies (6).

School bullying, as a negative life event, has been identified as an intentional aggressive behavior involving perpetrators and victims in school settings and mainly includes physical and verbal attacks and social exclusion (7). Numerous studies have found that the incidence of bullying among middle school students is as high as 16–36% (8, 9). Recently, school bullying has received great attention worldwide due to its negative impact on the physical and mental health of adolescents (10). Adolescents who e experience being bullied at school are found to be at an increased risk of poor academic performance, low self-esteem, anxiety, depression, and even self-harm behaviors (11, 12). Many previous studies indicated that bullying was one of the risk factors for NSSI and SA. For example, middle school students who had experienced being bullied were at a higher risk of suicide attempts [OR = 2.0, 95% CI (1.20–3.40)] (13) and NSSI [OR = 2.1, 95% CI (1.65–2.69)] in China (14). However, not all adolescents who have such experience engage in self-harm behaviors; thus, there might be some factors that mediate the relationship between school bullying and self-harm (15).

Many studies have demonstrated that depression and anxiety are important risk factors and predictors of SAs and NSSI (16, 17). It has also been found that depressive symptoms, psychological resilience (18), self-criticism, and stressful life events (19) are mediators of the relationship between bullying and NSSI, while loneliness, sleep problems, and depressive symptoms are mediators of the relationship between school bullying and SAs (20).

Academic stress, as an important environmental factor, is one of the major sources of stress for most students, especially middle school and high school students in China (21). During the COVID-19 pandemic, students have experienced greater academic stress than other populations due to the shift of the educational environment from campus to online, as well as changes in teaching methods (22). With great peer pressure, high academic expectations, and a large amount of homework, students have experienced greater academic pressure than before (23). Thus, academic stress has been regarded as an important factor affecting health-related issues, such as depression and anxiety, among Chinese adolescents (24, 25). Previous studies have shown that there was a significantly positive correlation between academic stress and anxiety/depression symptoms, and anxiety and depression were found, in turn, to be strong predictors of self-harm behaviors (26, 27). However, the mechanism underlying the relationship between academic stress and self-harm behaviors remains underexplored.

NSSI and SAs differ in the purpose of the behavior, i.e., whether there is suicidal intention. Therefore, we speculated that the association between bullying and NSSI might differ from the relationship between bullying and SAs. However, to our knowledge, there are few relevant studies that illustrate the difference between the bullying-NSSI link and the bullying-SA link using the same group of adolescents. Additionally, there is also a lack of studies clarifying the association and underlying mechanism between academic stress and NSSI or SAs. Thus, a better understanding of factors related to NSSI and SAs is important for the prevention and intervention of short-term or long-term impacts of these harmful behaviors. Additionally, as a stage of adolescence, middle school students must adapt to great changes in physical, emotional and social development. At the same time, they are also under great pressure because their academic performance determines whether they can enter high school (28). Because this change may result in psychological problems and affect their physical and mental health, it deserves our attention.

In the present study, we aimed to (1) explore the associations of academic stress and school bullying with self-harm behaviors among Chinese middle school students, (2) examine the direct effects of school bullying and academic stress and the indirect effects of anxiety and depression on self-harm behaviors, and (3) compare the different paths affecting NSSI and SAs. We also hypothesized that NSSI and SAs might be associated with academic stress and school bullying, and the outcome of NSSI or SAs might depend on the resulting psychological states of anxiety and depression. Hopefully, the findings of this study can provide evidence-based theories for clinicians and educators to perform effective interventions with adolescents.

## 2. Materials and methods

### 2.1. Participants and procedures

The present study adopted a cross-sectional design. Data were collected using cluster sampling, with participants recruited from a middle school in Changsha, China, from August 2021 to October 2021. The Chinese online platform “Wenjuanxing” was used to collect information and responses from all participants. Our study was an online survey, and the link was sent to the parents. After parents understood the purpose of the experiment in detail and agreed to participate, they were required to click the “Agree” button online, and then students participated in the survey and completed the questionnaire accompanied by parents. Participants were invited to respond to a questionnaire if they fulfilled the following criteria: (1) aged between 12 and 16, (2) in grades 7–9, and (3) fully understood the content of the questionnaire. The exclusion criteria were as follows: (1) refused to participate in the investigation, (2) engaged in both NSSI and SAs, and (3) did not complete the questionnaire carefully. This study was part of the China Depression Cohort Study (CDCS) and approved by the ethics committee of The Second Xiangya Hospital of Central South University.

### 2.2. Instruments

#### 2.2.1. Sociodemographic data

Sociodemographic data were collected using a self-designed questionnaire, which included age, gender, grade, family structure, history of smoking and drinking, and relationship with peers and family. The history of smoking and drinking was assessed using two dichotomous items: “Have you ever smoked one or more cigarettes a day?” And “Have you ever consumed alcohol once a week or more?”. The responses to both questions were “Yes” or “No.”

#### 2.2.2. School bullying

School bullying was assessed from the Olweus Bullying Victims Questionnaire (29) and has been modified. It was measured with the yes-no question, “Have you been bullied by other students at school this semester?” The forms of school bullying examined in this study included being teased, being threatened, rumor spreading, or being beaten, pushed, or hurt by one or more students in the school setting.

#### 2.2.3. Academic stress

Academic stress (26, 27) was measured with the question, “How much academic stress did you feel during the past semester?”, the answer to which was rated on a 5-point Likert scale (1 = no stress, 2 = relatively low stress, 3 = average stress, 4 = relatively high stress, and 5 = extreme stress), with higher scores indicating greater perceived academic stress. In this study, a score of 4 or 5 was defined as a high level of academic stress, a score of 3 indicated medium levels of academic stress, and 1 or 2 indicated low levels of academic stress.

#### 2.2.4. Anxiety and depression

The 9-item Patient Health Questionnaire (PHQ-9) (30) is a self-assessment scale that has been extensively used to measure depressive symptoms in the previous 2 weeks in different populations, including adolescents. All the items were measured on a 4-point Likert scale (0 = not at all, 1 = several days, 2 = more than 1 week, and 3 = nearly every day), and higher total scores indicated higher severity of depressive symptoms. The PHQ-9 scale had good reliability and validity in the Chinese population (30), with a Cronbach's alpha of 0.892 in this study.

The Chinese version of the General Anxiety Disorder-7 scale (GAD-7) (31) was used to measure anxiety symptoms during the last 2 weeks. The GAD-7 contains 7 items rated on a 4-point Likert scale (0 = not at all, 1 = several days, 2 = more than 1 week, and 3 = nearly every day). The GAD-7 has also been widely used and reported to have favorable reliability and validity (32); the Cronbach's alpha was 0.911 in this study.

#### 2.2.5. Self-harm behaviors

According to the diagnostic criteria in the 5th Diagnostic and Statistical Manual of Mental Disorders (DSM-5) (33), NSSI was assessed using a yes-no question: “Have you ever hurt yourself with the intention of suicide in the past 12 months, with the use of burns, cuts, blows, stabs and other means?” SA was assessed using an item in the MINI-International Neuropsychiatric Interview (M.I.N. I): “Have you ever attempted suicide?” The responses to both questions were either “yes” or “no.” The purpose of this study was to explore the independent influence of various factors on self-harm behaviors, such as SAs and NSSI. To control confounding variables and make the results more reliable, in the present study, we excluded those who had been engaged in both NSSI and SA behaviors.

### 2.3. Statistical analysis

SPSS 24.0 software and AMOS structural equation model statistical software were used for data analyses. First, sociodemographic characteristics and clinical variables were analyzed using descriptive statistics. Continuous variables are presented as the mean ± standard deviation, and categorical variables are presented as frequencies and percentages. A univariate logistic regression analysis was then performed to examine the associations between the variables with SA and NSSI, and variables with *P* < 0.01 were regarded as significant influencing factors. Finally, mediation analysis using the AMOS structural equation model was performed to examine the associations between school bullying, perceived academic stress, anxiety, depressive symptoms, SAs, and NSSI, with the 95% confidence interval (CI) of the indirect effect not including zero indicating that the mediation effect was present (*P* < 0.05).

## 3. Results

### 3.1. Descriptive statistics

A total of 1,313 Chinese middle school students (grades 7–9), including 609 boys and 704 girls, were enrolled in the study; they were 12–16 years of age (13.4 ± 0.7 years). The results showed that 487 students (37.1%) reported a high level of academic stress, and 164 students (12.5%) reported experiencing bullying at school. A total of 54 students (4.1%) reported NSSI, while 44 students (3.4%) reported SAs. The sociodemographic and clinical data are presented in Table 1.

**Table 1:** Demographic characteristics and clinical variables (*N* = 1,313).

		**Mean**	**SD**
Age		13.4	0.7
GAD score		2.3	3.9
PHQ score		2.8	4.5
		Frequency	Percent (%)
Gender	Male	609	46.4
	Female	704	53.6
Grade	7	429	32.7
	8	507	38.6
	9	377	28.7
Family structure	Two-parent family	1,206	91.9
	Non-two-parent family	107	8.1
Smoking	No	1286	97.9
	Yes	27	2.1
Drinking	No	1,271	96.8
	Yes	42	3.2
Academic stress	High	487	37.1
	Moderate	826	62.9
Relationship with peers	Low	16	1.2
	Moderate	302	23.0
	Good	995	75.8
Relationship with family	Poor	40	3.0
	Moderate	261	19.9
	Good	1,012	77.1
Experience of being bullied at school	No	1,149	87.5
	Yes	164	12.5
NSSI	No	1,259	95.9
	Yes	54	4.1
SA	No	1,269	96.6
	Yes	44	3.4

### 3.2. Univariate logistic regression analysis

The results of the univariate regression analysis of variables associated with NSSI and SAs are presented in Table 2. Anxiety [odds ratio (OR) = 1.23, 95% CI: 1.16–1.28], depression (OR = 1.20, 95% CI: 1.16–1.25), smoking (OR = 7.37, 95% CI: 2.84–19.09), drinking (OR = 6.27, 95% CI: 2.75–14.29), academic stress (OR = 2.20, 95% CI: 1.27–3.80), and school bullying (OR = 3.80, 95% CI: 2.11–6.89) were all significantly associated with NSSI. Anxiety (OR = 1.25, 95% CI: 1.19–1.31), depression (OR = 1.26, 95% CI: 1.20–1.31), academic stress (OR = 3.80, 95% CI: 1.20–7.25), and school bullying (OR = 2.76, 95% CI: 1.39–5.47) were all significantly associated with SAs. Furthermore, there was a significant positive association of anxiety and depressive symptoms with academic stress (OR = 1.11, 95% CI: 1.07–1.14; OR = 1.10, 95% CI: 1.06–1.12) and school bullying (OR = 1.10, 95% CI: 1.06–1.14; OR = 1.10, 95% CI = 1.06–1.13).

**Table 2:** Univariate regression analysis of variables associated with NSSI and SA.

		**NSSI**			**SA**		
		***P***	**OR**	**95% CI**	***P***	**OR**	**95% CI**
Age		0.929	1.00	0.67–1.45	0.264	1.28	0.83–1.97
GAD score		< 0.001	1.23	1.16–1.28	< 0.001	1.25	1.19–1.31
PHQ score		< 0.001	1.20	1.16–1.25	< 0.001	1.26	1.20–1.31
Gender	Male	Reference			Reference		
	Female	0.095	0.62	0.35–1.10	0.100	1.70	0.90–3.20
Grade	7	Reference			Reference		
	8	0.208	0.64	0.31–1.29	0.044	0.45	0.21–0.98
	9	0.526	0.81	0.43–1.54	0.116	0.57	0.29–1.15
Family Structure	Two-parent family	Reference			Reference		
	Non-two-parent family	0.761	0.86	0.34–2.21	0.816	0.88	0.31–2.52
Smoking	No	Reference			Reference		
	Yes	< 0.001	7.37	2.84–19.10	0.251	2.37	0.54–10.33
Drinking	No	Reference			Reference		
	Yes	< 0.001	6.27	2.75–14.29	0.177	2.31	0.69–7.78
Academic Stress	Low/moderate	Reference			Reference		
	High	0.005	2.19	1.27–3.80	< 0.001	3.81	2.00–7.25
School Bullying	No	Reference			Reference		
	Yes	< 0.001	3.81	2.11–6.89	0.004	2.76	1.39–5.47

### 3.3. Mediation analysis

**Figure 1:**
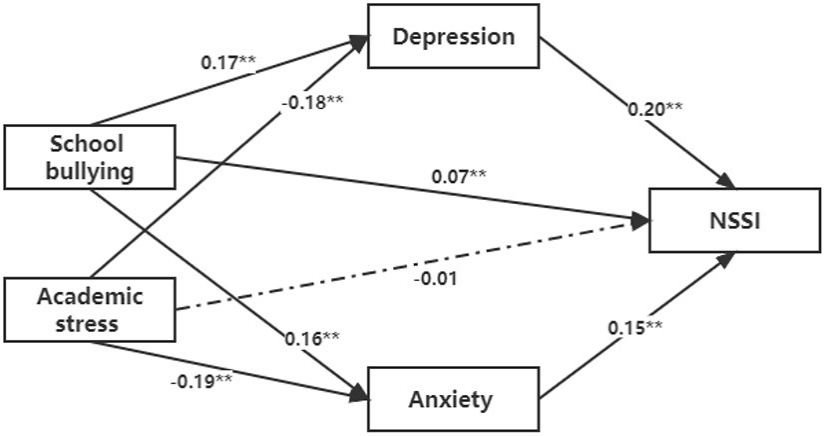
The mediation analysis on the relationship between school bullying and academic stress and NSSI (** *P* < 0.05; * *P* < 0.10).

**Figure 2:**
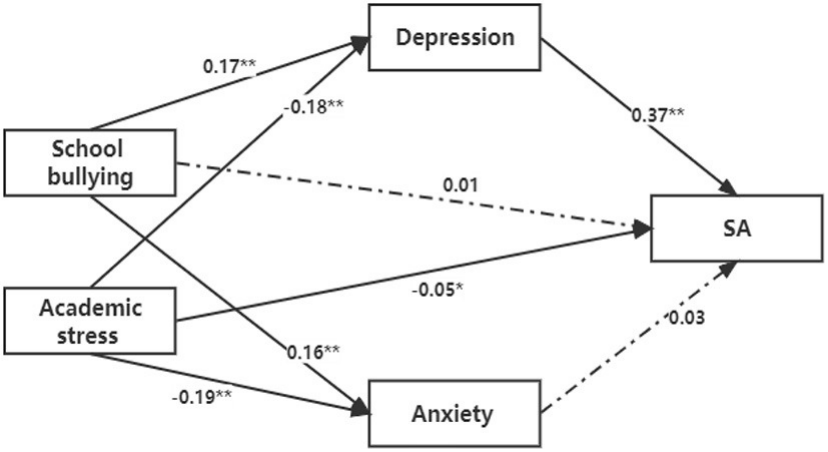
The mediation analysis on the relationship between school bullying and academic stress and SA (** *P* < 0.05; * *P* < 0.10).

The mediation analysis revealed potential mediation effects of anxiety and depression on the associations of academic stress and school bullying with SAs and NSSI among Chinese middle school students. The results showed that both depression and anxiety had a partial mediation effect on the association between school bullying and NSSI (*P* < 0.05), while both had a complete mediation effect on the relationship between academic stress and NSSI (*P* < 0.05) (see Figure 1). Depression was found to have a complete mediation effect on the relationship between school bullying and SAs (*P* < 0.05) and a partial mediation effect on the relationship between academic stress and SAs (*P* < 0.05). No mediation effect of anxiety was found on the association between academic stress and school bullying and SAs (see Figure 2).

## 4. Discussion

By investigating the relationship between school bullying, academic stress, anxiety, depression, and NSSI and SAs, this study revealed the direct and indirect effects of anxiety and depression using a relatively large sample of middle school students.

In this study, we found that self-harm behaviors were significantly associated with academic stress and school bullying, and anxiety and depression were associated with different self-harm behaviors (NSSI or SA). Both depression and anxiety had a partial mediation effect on the association between school bullying and NSSI, indicating that being the victim of school bullying is not only indirectly related to the risk of NSSI through the mediation of depression and anxiety (especially depression) but also directly associated with a higher risk of NSSI. Students who have been bullied at school may fail to seek help or develop appropriate social and coping skills, resulting in unhealthy emotional reactions, coping styles and self-harm behaviors (34). Notably, the present study showed that the effect of school bullying on NSSI was mediated by anxiety and depression, which needs to be considered when formulating intervention strategies.

Both depression and anxiety had a complete mediation effect on the relationship between academic stress and NSSI, suggesting that academic stress was only indirectly associated with the risk of NSSI through the mediation of depression and anxiety, especially anxiety. In the education context in China, the majority of teenagers strive to live up to their parents' expectations and prove their abilities by achieving higher grades (25). Thus, teenagers may experience considerable academic stress, which may lead to psychological or mental health problems, such as anxiety and depression (26). Some previous studies suggested that NSSI might be regarded as a way of emotional and stress venting, help seeking, self-punishment and avoidance among adolescents (35). Moreover, we also found that school bullying was more correlated with depression, while academic stress was more correlated with anxiety, which might be attributed to the greater connection between sadness/helplessness and bullying and the greater connection between worries/uncertainty and academic stress (18, 26).

According to the current findings, high levels of risk of NSSI were associated with high levels of school bullying and loneliness, and sensitivity analysis showed that fluctuations in emotional responses played a mediating role (36). This further validated NSSI's interpersonal model, namely, that negative interpersonal relationships significantly promote NSSI. NSSI can be used as a warning signal of experiencing bullying and expands the research on NSSI as a predictor of interpersonal stress (37). Academic and interpersonal relationships are the two most important stressors for students. Once problems occur, students are likely to be troubled by depression, anxiety and other emotions, which will lead to self-harm behaviors. Furthermore, adolescents who self-harm are often stigmatized, which may increase the risk of bullying and the experience of more interpersonal stress through increased negative emotions, which complement and reinforce each other (38).

We also found that depression had a complete mediation effect on the relationship between school bullying and SAs and a partial mediation effect on the relationship between academic stress and SAs. However, no mediation effect of anxiety was found on the association between academic stress and school bullying and SAs. This indicated that SAs were associated with school bullying through the mediation of depression only. As mentioned above, students who experience being bullied might fail to seek help due to their traumatic experience and negative emotions, which may result in being prone to despair, suicidal thoughts and self-harm behaviors (39). The present study also showed that academic stress was indirectly associated with SAs through the mediation of depression and directly associated with SAs. Students with greater academic stress might have been living in the expectations of their parents and teachers, with their own ambitions, as well as under peer pressure, which might lead to depression and even an increased risk of suicide when they fail to achieve their goals (40). Therefore, early identification of academic stress and unhealthy emotions and behaviors as well as timely interventions may be effective approaches to reduce suicidal behaviors among adolescents. It is also important to reduce SAs by timely detection and intervention of depressive symptoms in adolescents who are bullied and reduce NSSI by mitigating depression and anxiety in those under great academic stress. Therefore, the different mechanisms and significance of NSSI and SAs in this study have important guiding significance for the formulation of intervention strategies.

Many previous surveys involving Chinese middle school students have also reported that a large number of students displayed severe symptoms of anxiety and depression and were at a higher risk of suicide and self-injury (41). Furthermore, there was a significant positive association of anxiety and depressive symptoms with academic stress and school bullying. Students who had been bullied at school were likely to feel sad, hopeless, and fearful, might worry that similar things would happen again, and might be too afraid to tell their parents and teachers about the bullying, which affected the students' emotional state and led to serious adverse consequences such as NSSI or SAs (12). A high level of academic stress was also found to be associated with anxiety and depression and a risk factor for SAs and NSSI. As adolescents are psychologically and emotionally immature, they are more likely to be affected by academic stress (25). Thus, a relaxed and pleasant school environment, an effective supervision system, and trained teachers are needed. Students and their parents also need training in stress management skills and parenting styles, respectively, to maintain good mental health in adolescents.

The present study showed that a large proportion of Chinese middle school students (37.1%) reported high levels of academic stress; this was similar to that reported in another domestic study (34.1%) (42) but significantly higher than those found in some international studies (26.5–29.5%) (43). The reason may be that under the influence of Confucianism and other cultural values, Chinese parents and teachers tend to attach great importance to children's education and academic achievement, and adolescents are often required to invest time and energy in their studies, which, according to their parents, can change their lives and futures (44). The present study showed that 12.5% of the students reported experiencing being bullied at school, while some other studies reported different results (11.6–16.7%) (13, 15). These findings suggested that school bullying has become a common and serious problem among middle school students, which needs more attention and concern. In addition, 4.1% of the students reported NSSI, and 3.4% reported SAs, suggesting that self-harm behaviors have become a problem that cannot be ignored. Thus, it is important to understand the mechanism of the phenomena and develop targeted solutions.

### 4.1. Strengths and limitations

The present study has several strengths. First, the relationship between academic stress, school bullying and self-harm behaviors among Chinese middle school students was explored using a relatively large sample. Second, we examined the mediation effects of depressive and anxiety symptoms on the relationship between academic stress and school bullying and self-harm behaviors, which is more detailed than analyzing the pairwise association only. Third, to our knowledge, this was the first study examining the indirect and direct relationship between academic stress and suicide attempt, as well as the mediation effect of depression. Finally, we investigated the effects of academic stress and school bullying on NSSI and SAs separately, which displayed different results. Despite the strengths, there were still some limitations. First, the cross-sectional design precluded us from examining the causal relationships between the studied variables. Thus, longitudinal studies are needed to verify the findings of this study in the future. Second, academic stress, school bullying and self-harm behaviors were assessed using participant responses to questions; thus, the results might have been affected by recall bias and selection bias. In future research, more reliable and rigorous tools are needed for data collection, and multiple sources of information, including parents and teachers, are also needed. Finally, the participants in this study were from only one city, which might have limited the generalizability of our findings. The application scope and population of this study should be expanded in the future.

## 5. Conclusions

The present study found that associations involving academic stress, school bullying, self-harm behaviors, anxiety and depression are salient among adolescents. We also found a direct effect of academic stress and school bullying on NSSI and SAs, as well as an indirect effect of the risk factors through the mediation of anxiety and depression, indicating that depression played an important role in the development of NSSI and SAs among adolescents who had experienced great academic stress and school bullying. Overall, the results of this study provided data support to emphasize the importance of reducing academic pressure and creating a good school environment for Chinese adolescents to reduce negative emotions and behaviors as well as improve the physical and mental health of adolescents.

## Data availability statement

The raw data supporting the conclusions of this article will be made available by the authors, without undue reservation.

## Ethics statement

Written informed consent was obtained from the individual(s), and minor(s)' legal guardian/next of kin, for the publication of any potentially identifiable images or data included in this article.

## Author contributions

HuC, XW, and JZ conceived and designed the study. HaC, XCh, HT, YT, and JL participated in the acquisition of data. HG and XCa analyzed the data. HuC and HG drafted the manuscript. HuC, HG, XW, and JZ revised the manuscript. All authors read and approved the final manuscript.
